# Gastric Schwannoma Presenting as an Incidentaloma on CT-Scan and MRI

**DOI:** 10.4021/gr245w

**Published:** 2010-11-20

**Authors:** Menno H. Raber, Cathelijne M.P. Ziedses des Plantes, Robert Vink, Joost M. Klaase

**Affiliations:** a Department of General Surgery, Medisch Spectrum Twente, Enschede, the Netherlands; b Department of Radiology, Medisch Spectrum Twente, Enschede, the Netherlands; c Laboratorium Pathologie Oost Nederland, Enschede, the Netherlands

**Keywords:** Gastric schwannoma, S-100, Neurinoma, Neurilemmoma, MRI, CT, Immunohistology

## Abstract

A 67 year old female was referred because of an incidentaloma on CT-scan and MRI which showed a 5.0 cm large mass in the wall of the distal stomach. After an initial work-up which suggested a gastrointestinal stromal tumor (GIST), a partial gastrectomy with a Billroth II gastrojejunostomy was performed. The histological diagnosis was a schwannoma. Gastric schwannomas are rare tumors which comprise 0.2% of all gastric tumors and 4% of all benign gastric neoplasms with a peak of incidence in the 4th and 5th decade of life. Gastric schwannomas are usually asymptomatic, but can present with ulceration and/or gastrointestinal bleeding. Clinical, endoscopical, surgical, radiological and histological features of this case are described and the relevant literature is reviewed.

## Introduction

Peripheral nerve sheath tumors may arise from Schwann cells, perineural cells and fibroblasts. If a tumor originates from a Schwann cell, it is generally called a schwannoma, but may also be known as a neurinoma or a neurilemmoma. Schwannomas are benign, slow growing tumors and are generally found in the cranial vault. The most common localization if found extradural, is in association with large nerve trunks. The gastrointestinal (GI) tract is a relatively rare localization for schwannomas, but if found there the most common localization is the stomach. Gastrointestinal schwannomas are usually asymptomatic. The differentiation between a schwannoma and other submucosal tumors of the gastrointestinal tract, like gastrointestinal stromal tumor (GIST), can be difficult pre-operatively. The microscopic findings with aid of immunohistochemistry (S100 positivity) can however make a definite diagnosis.

We present a 67-year-old woman with a gastric schwannoma manifesting with abdominal soreness and dyspepsia. The diagnosis was histologically confirmed after surgery. The relevant literature is reviewed.

## Case Report

This 67-year-old woman was referred to our center after a mass was detected in the gastric wall on CT-scan and Magnetic resonance imaging (MRI) at a commercial imaging centre. Anamnestically she sometimes had vague abdominal soreness and periodic dyspepsia which she managed with over the counter antacids. There were no other complaints and there was no weight loss.

**Figure 1:**
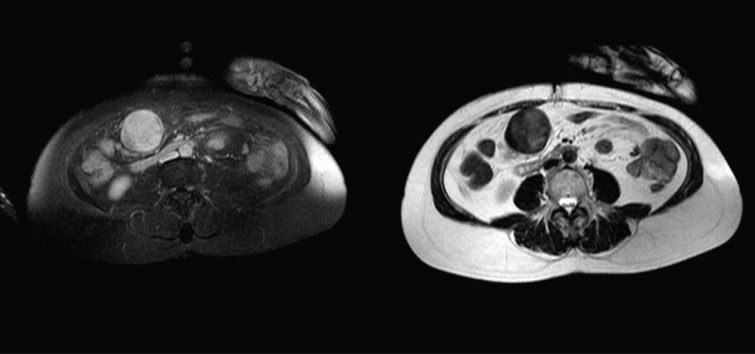
Left a contrast enhanced T1 weighted image with an overall inhomogeneous high signal pattern; Right a T2 weighted image with a low to intermediate signal pattern.

**Figure 2:**
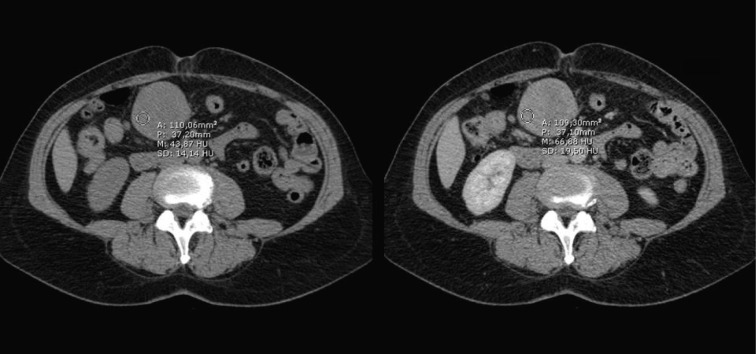
CT-scan showing on the left a non contrast image of the lesion and on the right a post contrast image showing slight enhancement, depicted as the drawn circle with the mean Hounsfield units.

The MRI and CT-scans, made at the commercial imaging centre, showed on the MRI a 5 cm, sharply demarcated mass adjacent to the greater curvature of the stomach. The overall signal pattern was inhomogeneous high on post gadolinium T1 weighted images (pre-gadolinium images were not available) and low to intermediate on T2 weighted images (Fig. 1). On CT the mass was inhomogeneous with a small rim of high density which probably was stomach contents and showed very subtle venous enhancement (Fig. 2).

**Figure 3:**
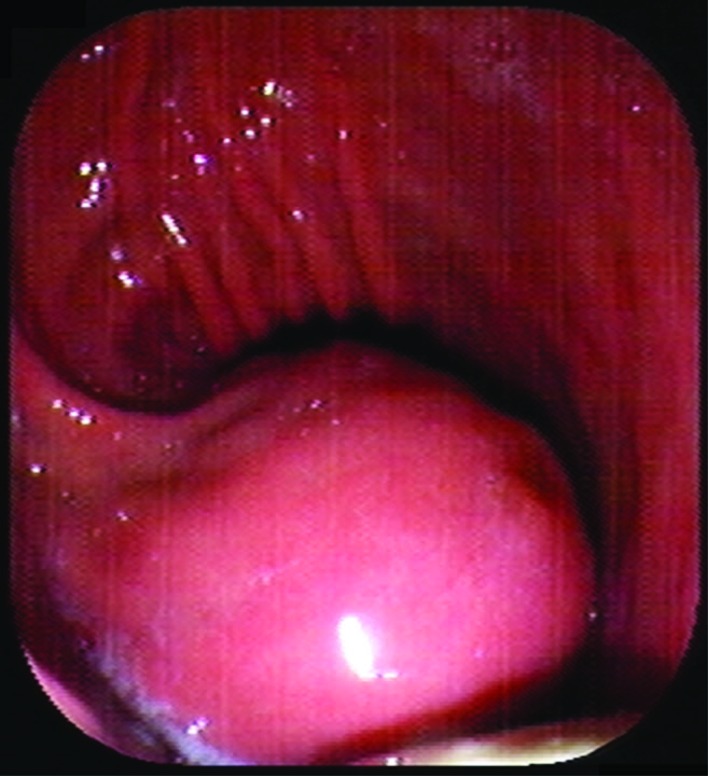
An exophytically growing, submucosal mass is seen in the stomach during endoscopy.

An endoscopic ultrasound was performed (Fig. 3) and fine needle aspirations were taken. It was however not possible to make a definite pre-operative diagnosis based on this material. The most likely diagnosis was considered to be a gastrointestinal stromal tumor (GIST).

**Figure 4:**
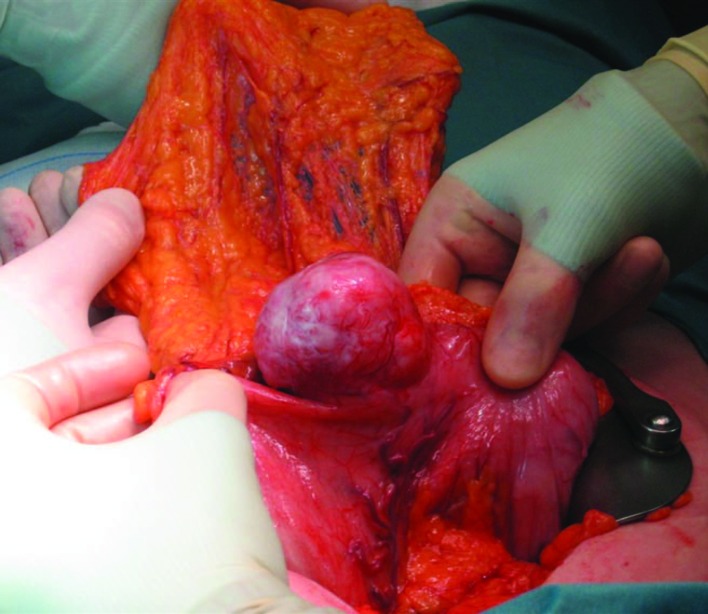
Macroscopic inspection during surgery showed a tumor with a diameter of 7 cm in the distal stomach.

**Figure 5:**
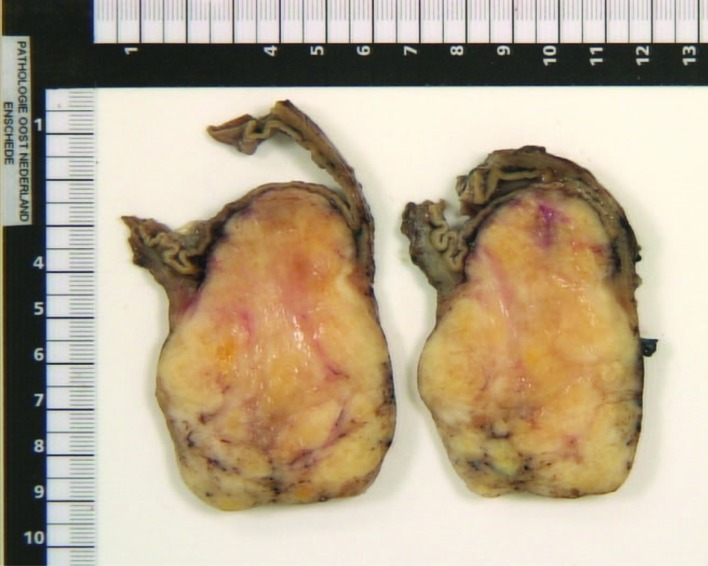
Cross section of the tumor, revealing an off-white mass with an intact overlying mucosal layer.

**Figure 6:**
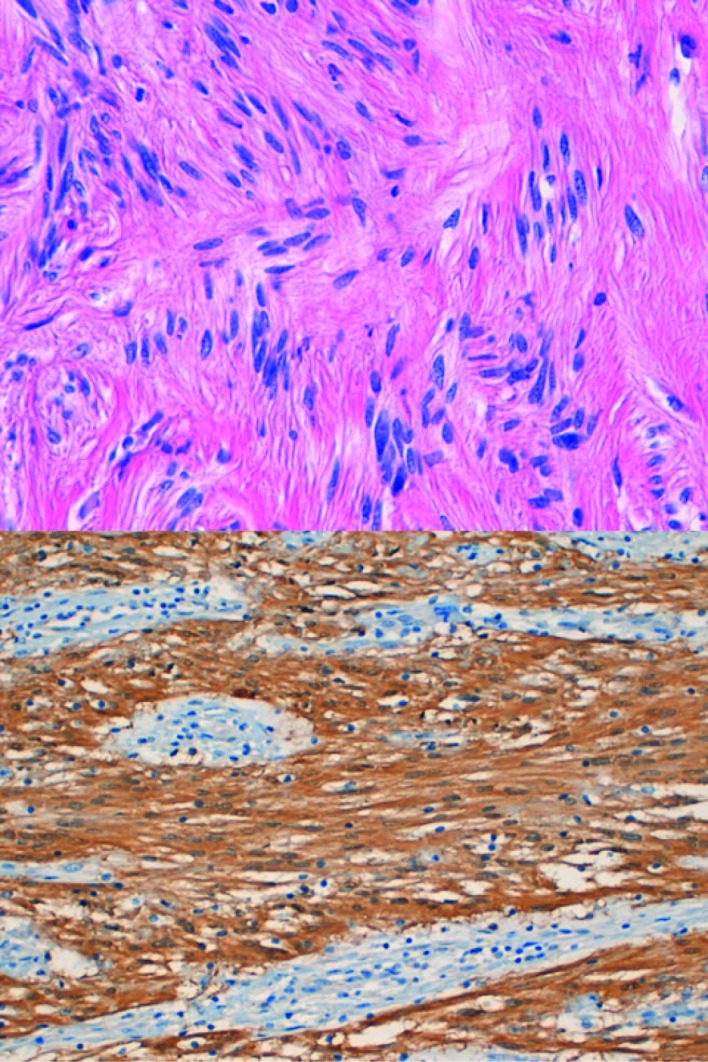
Upper: Schwannoma (Spindle cells with nuclear palisading); Lower: diffuse S100 positivity in Schwann cells.

**Figure 7:**
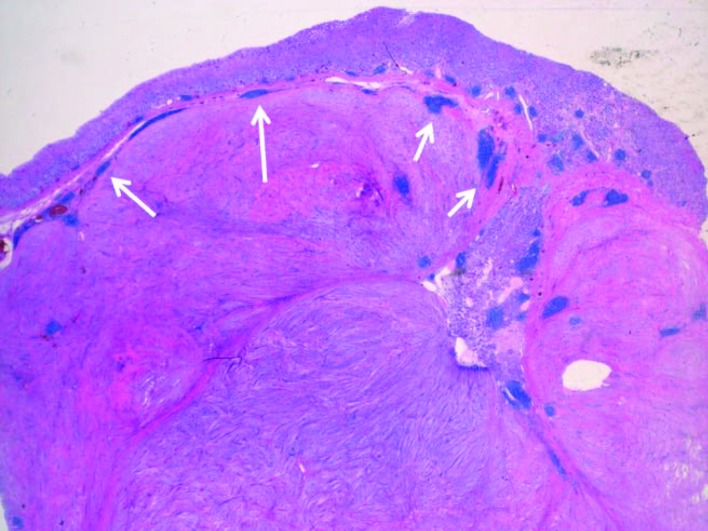
Lymphoid infiltrates (see arrows) at the periphery of the lesion are a common and characteristic feature.

A partial gastrectomy with a Billroth II gastrojejunostomy was performed in order to remove the tumor (Fig. 4). Macroscopic inspection showed a tumor with a diameter of 7 cm. The tumor was whitish in color, and had a firm consistency (Fig. 5). Microscopy showed a lesion consisting of spindle cells and Schwann cells. The nuclei are fusiform and show little variation in size and shape. Mitoses are infrequent. There is some nuclear palisading (Fig. 6). Typical lymphoid infiltrates at the periphery of the lesion were seen (Fig. 7). Immunohistochemistry revealed diffuse positivity for S-100 protein (Fig. 6). There was negativity for CD 34 and CD 117.

The postoperative period was uneventful and the patient was dismissed from the hospital after five days.

## Discussion

Schwannomas are benign, slow growing tumors arising from Schwann cells. In the gastrointestinal (GI) tract they are infrequently found with the stomach as the most common localization. Schwannomas comprise 0.2% of all gastric tumors and 4% of all benign gastric neoplasms [1]. The peak incidence is in the 4th and 5th decade of life [1]. Gastrointestinal schwannomas are usually asymptomatic for a long time, like in the case reported above. When symptomatic, the most common findings are ulceration and/or gastrointestinal bleeding [1-5].

The differentiation between a schwannoma and other gastrointestinal mesenchymal tumors, like GIST, can be difficult pre-operatively. Most gastric schwannomas show CT features of hypodense, well-demarcated and homogeneous tumors with contrast enhancement, like the case was in our patient as well [6]. Endoscopic ultrasonography usually shows homogenous, submucosal tumors, which sometimes grow exophytically [6]. This feature can be seen both with GIST’s and schwannomas. Endoscopic tissue biopsies in most cases yield inconclusive results because mucosal abnormalities are rarely observed in these mainly submucosal tumors [6]. MRI findings of spinal and cranial schwannomas are well described in literature. MRI features of gastrointestinal schwannomas seem to be similar. Most tumors are low to isointense on T1-weighted images and isointense to high intense on T2-weighted images [7, 8]. There was no pre-gadolinium scan available, which explains the high intensity on T1-weighted images, because these were post-gadolinium. The discrepancy in signal intensity between our T2-weighted images and those described in literature might be caused by the presence of melanin in the schwannoma.

Gastrointestinal schwannomas have an average size of 6 - 7 cm but range between 0.5 and 14 cm. Typical features of gastric schwannoma on gross pathology are submucosal localization with an intact mucosal layer. Schwannomas arise in the submucosa and muscularis propria [4, 6]. They may appear encapsulated, but usually interdigitate with the surrounding stromal cells. Lymphoid infiltrates at the periphery of the lesion are a common and characteristic feature (Fig. 6). The lesion consists of spindle cells and Schwann cells, lying in bundles of variable thickness or more loosely woven. The nuclei are fusiform and usually show little variation in size and shape. Significant nuclear atypia, considered to be degenerative in nature, can however occur. Mitoses are infrequent. Nuclear palisading can occur (Fig. 7) [9]. Immunohistochemistry consistently reveals diffuse positivity for S-100 protein (Fig. 7) [6]. The most common differential diagnosis for gastrointestinal schwannoma is a GIST. Most GISTs express CD117, CD34, DOG-1 and caldesmon, whereas a schwannoma does not express these markers [10, 11]. GISTs are believed to originate from Cajal cells, whereas schwannoma are thought to originate from Schwann cells [4, 10]. Definite diagnosis can be made based on (immuno-) histological findings.

In conclusion, Gastric schwannoma is an uncommon type of gastric neoplasm. It is usually asymptomatic, but can present with gastrointestinal bleeding or a palpable mass. Definite diagnosis can only be made through histopathological investigation. This means that if discovered, most schwannoma are surgically resected to exclude other diagnosis like GIST. Surgical resection is therefore the definite treatment.
